# Compact acceleration of energetic neutral atoms using high intensity laser-solid interaction

**DOI:** 10.1038/s41598-017-04152-3

**Published:** 2017-06-20

**Authors:** Malay Dalui, T. Madhu Trivikram, James Colgan, John Pasley, M. Krishnamurthy

**Affiliations:** 1 Tata Institute of Fundamental Research, 1 Homi Bhabha Road, Colaba, Mumbai, 400 005 India; 2 Department of Physics, Lund University, P.O. Box 118, 221 00 Lund, Sweden; 3 Los Alamos National Laboratory, Los Alamos, NM 87545 USA; 4 York Plasma Institute, University of York, Heslington, York YO10 5DQ, UK; 5 TIFR Centre for Interdisciplinary Sciences, 21 Brundavan Colony, Narsingi, Hyderabad 500 075 India

## Abstract

Recent advances in high-intensity laser-produced plasmas have demonstrated their potential as compact charge particle accelerators. Unlike conventional accelerators, transient quasi-static charge separation acceleration fields in laser produced plasmas are highly localized and orders of magnitude larger. Manipulating these ion accelerators, to convert the fast ions to neutral atoms with little change in momentum, transform these to a bright source of MeV atoms. The emittance of the neutral atom beam would be similar to that expected for an ion beam. Since intense laser-produced plasmas have been demonstrated to produce high-brightness-low-emittance beams, it is possible to envisage generation of high-flux, low-emittance, high energy neutral atom beams in length scales of less than a millimeter. Here, we show a scheme where more than 80% of the fast ions are reduced to energetic neutral atoms and demonstrate the feasibility of a high energy neutral atom accelerator that could significantly impact applications in neutral atom lithography and diagnostics.

## Introduction

Generating and analyzing a beam of high energy neutral atoms is a challenge that is important for many technological applications^1–3^. In lithographic applications, high energy neutral atoms result in higher finesse structures than those produced with charged particle beams^4^. High energy hydrogen atom beams play an important role in Tokamak diagnostics^5^. The methods used to accelerate neutral atom have seen very little progress until quite recently^6–8^.

The conventional techniques involve an ion source, accelerating columns and an efficient neutralizer, which employs either electron detachment (in case of negative ions) or electron capture (in case of positive ions) in a collision cell to convert ions to neutral atoms^9, 10^. Charge changing atomic collisions lead to beam straggling and beam divergence that reduce the beam current delivered to the target^11^. Decreasing areal density (product of gas density and collision cell length) and a more efficient neutralization process can lead to a better emittance of the neutral atom beam.

The charge of a positively charged projectile can be reduced either by capturing a bound electron from a target atom (in a collision cell) or by capturing a free electron^12^. The capture cross-section increases as the binding energy of the electrons that are captured from the target atom becomes smaller^13^. If the velocity of the projectile is close to the orbital velocity of the bound electron in the target atom, the electron capture cross-section increases. There are three dominant routes for free electron capture by an atomic ion^12^: (a) radiative recombination, (b) dielectronic recombination and (c) three-body recombination. In radiative recombination, the free electron capture is accompanied by radiation emission. The cross-section for this process is very low. Even for very highly charged ions (for instance, Ar^18+^) and sufficiently low electron temperature ($\simeq 10$ eV), the cross-section is about 10^−29^ cm^2^ ^13^. Moreover, with increasing electron energy, the cross-section decreases dramatically. For Ar^18+^, the cross-section is expected to decrease by about five orders of magnitude when the electron temperature increases from 5 eV to 10 eV^14^. Dielectronic recombination is the process by which the capture of a free electron leads to excitation of a bound electron in the host ion. The charge reduction is a two-step process whereby the de-excitation of the excited host ion in the second step leads to a charge-reduced system. Auto-ionization can deter the charge reduction. Since strong electrostatic fields are generated in high density plasmas, enhanced auto-ionization reduces the recombination rates. Three-body recombination occurs when the electron density is high such that two electrons simultaneously interact with the atomic ion and one of the electrons is captured while the other (scattered) electron carries away the excess momentum.

In intense laser produced plasmas, it is possible to efficiently generate a burst of low energy electrons of high brightness. Since electrons move faster than ions, they expand beyond the region of laser focus and can alter the recombination and charge transfer dynamics. Recently this was exploited to accelerate neutral atoms up to MeV energies^8^. Nano-clusters exposed to intense laser pulses leads to a very strong ionization. The electrons exploding from the clusters excite a large fraction of clusters beyond the focal volume to their Rydberg states. Coulomb exploded ions that traverse the Rydberg excited atomic clusters, where the charge transfer rates are orders of magnitude higher, undergo efficient neutralization. In just a few millimetres away from the laser focus almost 95% of the ions were observed to become neutral. While this proved the use of laser plasmas for enhanced charge transfer, there are two problems with this scheme that need attention for improving the generation of neutral beams. (a) Ion acceleration is achieved by Coulomb explosion of nano-clusters and the ion yields (consequently neutral atom yields) are orders of magnitude smaller than ion emission obtained from solid targets. (b) Ion emission is nearly isotropic and consequently the neutral-atom generation is also nearly isotropic. Ion acceleration from solid targets seems therefore to offer a more promising platform for energetic neutral atom generation. The interaction of high-intensity ultrashort laser-pulses accelerates ions along the target-normal direction by the target-normal-sheath-acceleration (TNSA) mechanism^15–19^. A high energy neutral atom source with the same emittance characteristics seen in ions generating from intense laser-produced plasma^20, 21^, would seem out of reach of conventional charge transfer methods of neutral atom generation. Charge transfer neutralization of ions accelerated in TNSA by using a gaseous medium is a possibility that has been demonstrated recently^22^. This method uses a separate gaseous cell for neutralization. In general cross-sections for charge transfer reactions are larger and the neutralization is efficient and the only drawback can be an increase in beam divergence, especially if highly charged ions are to be neutralized. Electron-ion recombination process have lower cross-section but have the advantage of not increasing the beam divergence. Three body recombination can be important in high density plasma and can be used as an alternative scheme of neutralization. A scheme where both ionization and neutralization can be combined in the laser produced plasma itself may be a more compact way to generate neutral atom beams. The present work shows a successful scheme that achieves neutralization using recombination in the plasma generated by the same pulse which is responsible for acceleration of ions.

**Figure 1:**
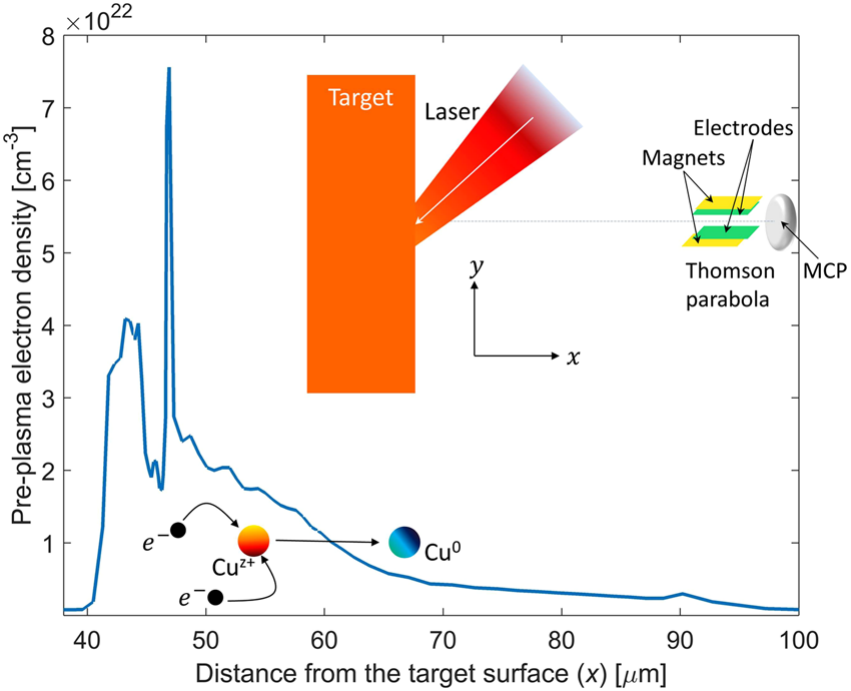
Neutral atom acceleration scheme: Intense laser-solid interaction produces strong transient fields and accelerates ions from the target surface. The pulse pedestal generates a pre-plasma (the electron density is shown here) and ions traversing through the pre-plasma undergo electron recombination, dominated by three-body recombination, to become neutral atoms with little change in momentum. Inset shows the experimental geometry and the location of the Thomson parabola for characterization.

The temporal profile of any intense femtosecond laser pulse does not normally resemble a delta function. Instead, in most laser systems (including ours), the pulse possesses a pedestal of much longer time duration before and after the ultrashort femtosecond part. This pedestal, though the intensity is one part of a million (of the peak), is long enough to produce significant volumes of plasma from a solid surface by multi-photon ionization and consequent cascade ionization^23^. This pre-plasma can be exploited as the neutralizer. Thus, our neutral atom accelerator involves three steps, as illustrated in Fig. 1. First, a pre-plasma is created by the pedestal of the laser pulse. Second, the main pulse resonantly excites a high amplitude electrostatic wave in the critical density layer resulting in a strong charge-separation field. The ions are accelerated in the capacitor-like transient sheath-electric-field^19, 24–26^. Third, the accelerated ions capture free electrons as they traverse in the pre-formed plasma and the electron-ion recombination results in formation of accelerated neutral atoms. Here, we demonstrate that under optimal conditions three-body recombination can be dominant, leading to ion-to-neutral conversion efficiency of greater than 80%. We show an acceleration of the order of 10^18^ g (*g* is the acceleration due to gravity at the Earth’s surface) in a length scale of a few hundred micrometers using high-intensity laser-solid interactions.

## Results and Discussion

**Figure 2:**
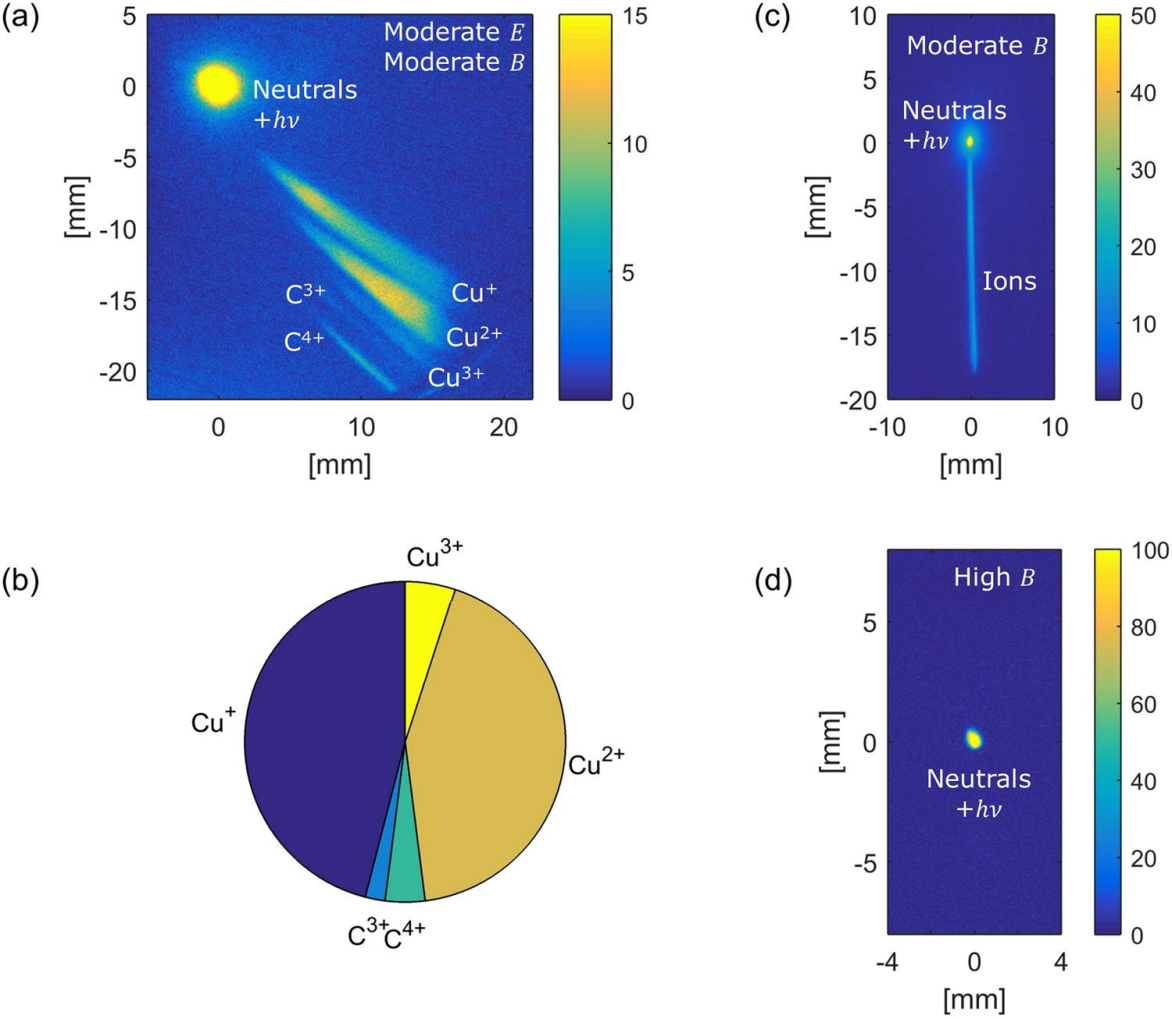
Thomson parabola spectrograms: (**a**) Shows a typical *q*/*m* resolved TP-spectrogram; (**b**) is a pie chart of the population of different *q*/*m* as derived from the TP-spectrogram. (**c**) Shows the ion trace when only magnetic field (of moderate strength) is used in the field region of the TP to deflect the ions and (**d**) is the scenario when a high magnetic field is applied to deflect all the ions from the detector. All the measurements are taken at a defocused position (*w* ≈ 50 *μ*m; *I* ≈ 10^17^ W/cm^2^).

**Figure 3:**
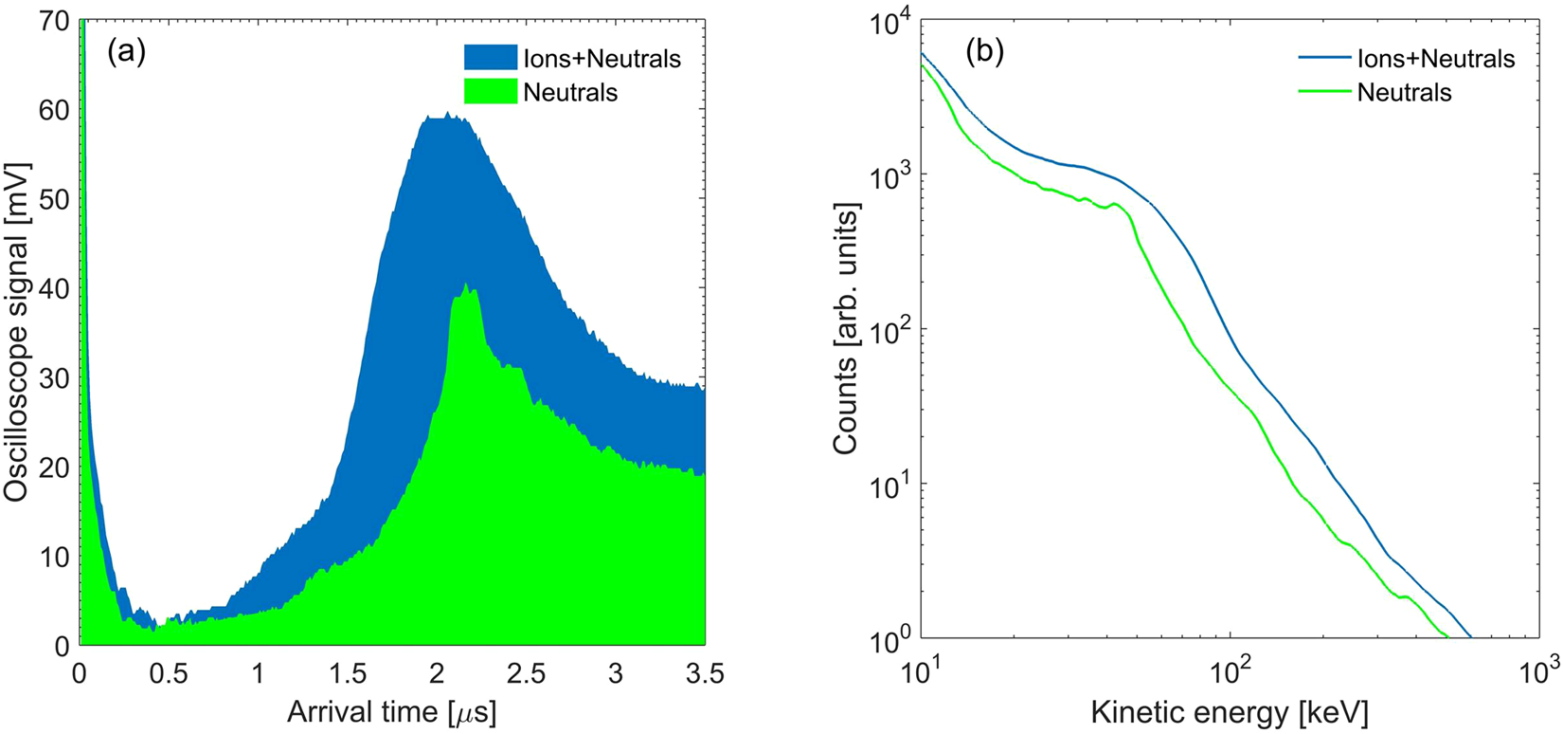
Arrival time profile and kinetic energy spectra: (**a**) The arrival time spectra of Cu atoms (the TP is operated with high deflection fields to push ions out of the detector; green trace) and Cu ions (no deflection fields; blue trace). (**b**) Shows the kinetic energy spectra of all Cu (ions and neutrals) and only the Cu neutrals as derived from the arrival times. The measurements are taken at a defocused position (*w* ≈ 50 *μ*m; *I* ≈ 10^17^ W/cm^2^).

The experimental setup is shown in Fig. 1. A Thomson parabola (TP) and arrival-time measurement techniques were simultaneously employed to detect and characterize the accelerated neutral atoms (look in the Methods section for details). In a TP spectrometer, ions traverse through a region where Electric and Magnetic fields are applied perpendicular to the traverse direction and the ions disperse in a parabolic path depending on the charge/mass (*q*/*m*). A position sensitive detector is used to record the splat position of the ion and record the parabolic trace that ions of different energy form for a given *q*/*m*. Figure 2a shows a TP image where the parabolic trace of different ions can be seen. The dispersion from the center, where neutrals or photons are recorded, gives the ions energy. Figure 2b shows a pie chart of the detected ionic species (integrated counts). We found that more than 90% of the detected ions are Cu. To detect neutral atoms, we have used only the magnetic field of the TP; sample data is illustrated in Fig. 2c and d. The ions are dispersed into a straight line (shown in Fig. 2a) when only a magnetic filed (of a moderate strength of 100 Gauss) is applied. Neutral atoms and photons, unaffected by this field, constitute the central bright spot. Then we increase the field strength (high B) such that all the ionic constituents are deflected out and the TP-spectrogram is left only with the central bright spot (Fig. 2d). Photons and heavier atoms reach the detector at different times; hence at this configuration an arrival time measurement can provide information about the neutrals. Figure 3a shows one of such measurement (with and without the highest deflection field). The contribution from photons marks the starting time (few ns); and the heavier neutral atoms reach the detector on a *μ* s time scale. The kinetic energy spectra as derived from the arrival time spectra is displayed in Fig. 3b with the assumption that all the atomic constituents are Cu. This assumption is valid as our measurements show that the majority of the emitted ionic species are of Cu (Fig. 2b). We have recorded on an average ~10^11^ accelerated neutral Cu atoms per laser pulse. The ion divergence was measured and we infer that the half-angle at half-maxima of the transverse spatial extent of the neutrals (which would be same as in ions) is about 15–20° at the source. The maximum acceleration with 150 mJ laser pulse is found to be approximately 1 MeV.

**Figure 4:**
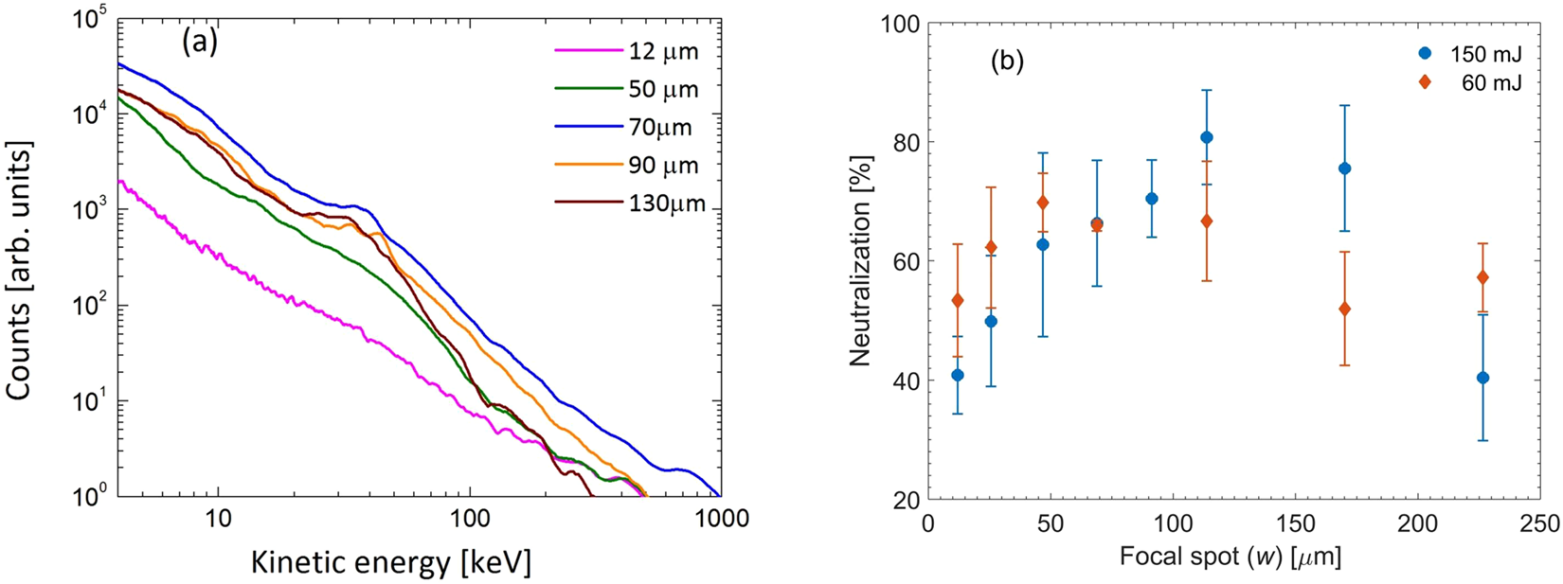
Characterization of accelerated neutral atoms. (**a**) Shows the kinetic energy spectra of the neutral Cu-atoms, and (**b**) is the neutralization fraction as derived from the arrival time signal for two distinct laser pulse energies at different defocused focal spots (*w*) as derived from arrival time profiles.

In our quest of optimal conditions for neutralization, we find that the most effective method is to move the target towards the focusing parabolic mirror and defocus the laser focal spot. The laser spot size on the target (*w*) increases with increasing defocusing. Figure 4a shows the energy spectra of the neutral atoms with laser defocusing as derived from arrival time signals when 150 mJ pulses (the corresponding intensity when *w* ≈ 12 *μ*m is ≈10^18^ W/cm^2^) are incident on the target. The ion acceleration appears to increase with the focal spot and the smallest spot size is not necessarily optimal for highest acceleration. The total neutralization fraction (energy integrated) as a function of the laser focal waist size is shown in Fig. 4b for two separate input laser energies. We notice that the optimum neutralization tends to shift towards lesser defocusing (lower *w*) for lower energy pulses. Increasing the focal spot size not only decreases the laser intensity, but also lowers the intensity of the pulse pedestal responsible for pre-plasma generation. A lower intensity pre-plasma decreases the electron density but more importantly it decreases the pre-plasma electron temperature^27^. The cold electron temperature is expected to scale linearly with the laser intensity^28^. Therefore, a tenfold increase in spot size will bring in a 100 fold decrease in both focused laser intensity and electron temperature. The cold electron temperature of the plasma (at *w* ≈ 12 *μ*m) in our experiments is about 300 eV^29^ and so, in the defocused condition, the pre-plasma temperature is expected to be about a few eV or less. Beyond the optimal defocusing the pre-pulse level goes below the pre-plasma formation threshold. The main pulse intensity also becomes low for effective ion acceleration. Hence, both the ion acceleration and the neutralization are diminished. Defocusing thus brings about two crucial changes: (a) the electron temperature of the pre-plasma is lowered dramatically with much smaller changes in electron density, and (b) the average charge states of the accelerated (by the main pulse) Cu ions are expected to be lower as the laser pulse intensity is decreased. The shift in the optimum defocusing for neutralization from lower energy pulses also suggests that the recombination dynamics is heavily dependent on the pedestal of the laser pulse.

**Figure 5:**
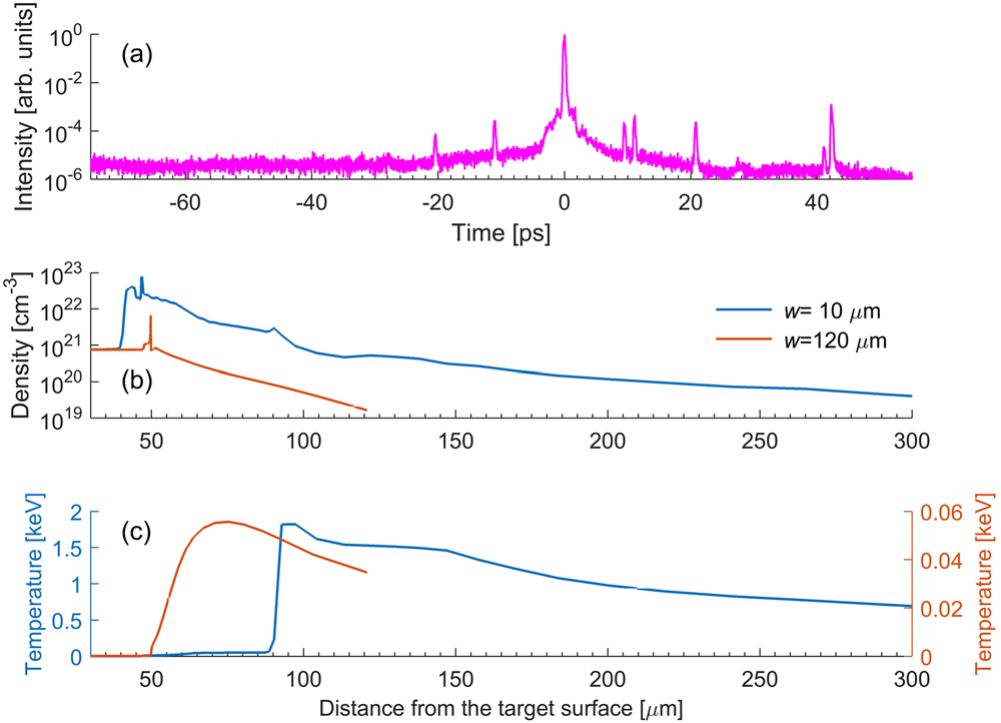
Temporal profile of the laser pulse and pre-plasma parameters: The electron density and temperature for two different focal waist conditions are generated using the HYADES 1-D radiation hydrodynamics code incorporating SESAME EOS and a multigroup radiation diffusion approximation. The intensity is increased as per the measured pre-pulse profile in (**a**) from about −100 ps to −1 ps and the resulting pre-plasma electron density and temperature vs. distance from the target initial surface is computed as shown in (**b**,**c**) respectively.

The experiments were carried out in a vacuum chamber maintained at ≈10^−5^ mbar. Since the mean free path of the electrons is more than 10 meters (compared to ion traversal length of about a meter), charge transfer collisions of the ions with the background gas can be neglected. Their contribution to the neutralization is computed to be less than 1%. To understand the neutralization process, we first need information on the pre-plasma profile in which the ions traverse before reaching the detector. We use the HYADES one dimensional radiation hydrodynamics simulation code to obtain the electron density and temperature profiles of the pre-plasma as a function of the distance away from the target-front surface (set at *x* = 0). The simulations take the measured laser pre-pulse (Fig. 5a) and employ a lagrangian radiation hydrodynamics solver incorporating multi-group radiation diffusion and a flux limited diffusion model for electron-thermal conduction. Figure 5b and c show the results for two different defocusing conditions used in the experiments labelled by the focal waist (*w*). The computations show peaks in electron density due to the formation of shock waves driven by the laser pre-pulse leading to a rarefaction wave into the target.

As ions traverse this plasma profile, neutralization occurs and to fully capture the charge reduction dynamics, we have used the ATOMIC code developed at the Los Alamos National Laboratory, USA. ATOMIC is a multi-purpose plasma kinetics code^30, 31^, that is used for modelling local thermodynamic equilibrium (LTE) and non-LTE plasmas, and can predict ionization balances, opacities and emissivities for a wide range of plasma conditions. The code takes as input atomic structure and collision data from the Los Alamos suite of atomic physics codes (for an overview, see ref. 32). In this case, the CATS code^33, 34^, was used in configuration-average mode to generate structure data and electron-impact excitation cross-sections for all ionization stages of Cu. The GIPPER code^32, 35^, was then used to generate all possible ionization cross-sections. ATOMIC was run in steady-state non-LTE mode^36^ (assuming zero radiation temperature) and used the electron temperature, density values and the initial average charge of the ions starting from the surface of the solid target produced by HYADES as input, resulting in predictions of the average ionization and charge distribution at each set of plasma conditions. Convergence tests with respect to the number of configurations included for each ionization stage were performed to ensure that the resulting average ionization predictions are converged. Since propagation of the HYADES code to long distances was computationally prohibitive, the electron temperature and density from Fig. 5 were extrapolated using an exponential scaling.

**Figure 6:**
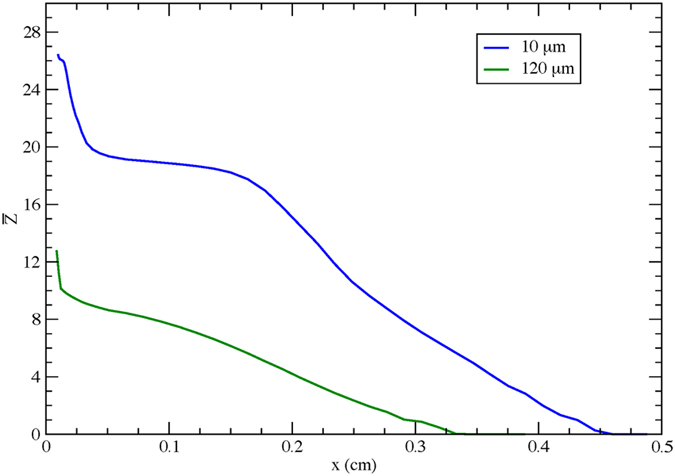
Evolution of the average charge state. Change in the average charge state ($\bar{Z}$) of the Cu ions as they propagate through the pre-plasma, away from the target surface. The pre-plasma density and temperatures computed by the HYADES code (see Fig. 5) are used to compute the electron recombination rates using the ATOMIC code.

In Fig. 6 we show the change in the average charge state ($\bar{Z}$) of Cu ions computed as a function of the distance traversed from the target surface for the focal waists of 10 *μ*m and 120 *μ*m. We find that, for both cases, the ions eventually recombine to form neutral atoms (corresponding to $\bar{Z}=0$). At the larger focal waist (*w* = 120 *μ*m) the ions start with a lower average charge and the overall recombination rates are larger, resulting in faster neutralization. In both of these cases, at any given distance the charge state is much lower with the larger focus than at the tight focus condition and so the neutralization fraction is expected to be larger with the larger focal waist. Although the ATOMIC code includes all possible recombination processes, we find that three-body recombination plays a dominant role, but that dielectronic recombination is also quite important. It is important to understand if the decrease in charge state or the lowering of the electron temperature has a larger role in neutralization. We note that while the average charge state is decreased by a factor of 2, the electron temperature is decreasing by about two orders in magnitude. Three-body recombination, which is the dominant process, depends more strongly on electron temperature than on charge states. Since the decrease in electron temperature is larger with increasing focal waist, the increase in neutralization with increase in focal waist appears to be due to the lower pre-plasma electron temperature. We also note that it is very difficult to fully compute the neutralization fraction found in the experiments due to many issues. For example, we will need to know more precisely the charge histogram at the front surface as the focal waist is changed. We also realize that apart from the pulse pedestal, the main pulse also changes the electron density and electron temperature profile. 2-D effects will play a role in the hydrodynamics and these will also affect the neutralization process. The pre-plasma electron temperature plays a crucial role in increasing the rate of three-body recombination and dielectronic recombination at larger focal waists. A well-defined ps pulse for pre-plasma generation and an fs pulse for ion acceleration can be used to establish the underlying process more precisely. A laser with a better pre-pulse contrast is mandatory for such experiments. However, the results described in this article demonstrate that tailoring the pre-plasma by means of defocusing is a viable option for increasing the neutralization fraction and thereby demonstrating a compact neutral atom accelerator.

An ion beam generated by the intense laser plasma has an intrinsic divergence. Angular divergence of the ions emitted from the front-side of the target is higher compared to ions accelerated from the rear side of a thin foil. Neutralization by electron-ion recombination does not significantly increase the ion beam divergence and is preferable. Studies have shown that ion divergence can be controlled by shaping the target surface^19, 37, 38^. As the ballistic propagation of ions is unaltered by the neutralization process, a shaped surface is expected to produce neutral beams with low angular divergence following the method described in this paper. Furthermore, the possibility of adopting this neutralization method for the ions generated at the rear side of a thin foil is likely to generate a less divergent neutral atom beam.

In summary, a compact neutral atom acceleration technique yielding a beam of neutral atoms with more than 80% neutralization efficiency from a laser-produced plasma has been experimentally realized. Acceleration up to an MeV energy has been achieved by this scheme (the corresponding acceleration is ~10^18^ *g*). The neutral atom yield as well as the ion-to-neutral conversion efficiency has been seen to increase with laser defocusing. The neutral atom acceleration process involves two steps. First, ions formed at the target surface are accelerated in the sheath electric field and second, electron recombination, dominated by three body recombination, results in charge reduction and neutralization without any significant change in momentum. The emittance of the neutral atom remains the same as the ions and the neutral atoms emanate from a spot of approximately 100 *μ*m diameter (corresponding laser intensity is of the order of 10^16^ W/cm^2^), expanding normal to the target in a 15° cone.

## Methods

The experiment was performed at the Tata Institute of Fundamental Research, Mumbai, India, using the high intensity short pulse laser system. In the experiment 800 nm, 40 fs, *p*-polarized laser pulses from a CPA-based^39^ Ti:sapphire laser system were employed. This system is capable of delivering light pulses at a repetition rate of 10 Hz. The laser pulse was focused on the target using a couple of guiding mirror and an off-axis parabolic mirror at an incidence angle of about 40°. A 5 cm × 5 cm × 5 mm Cu substrate was used as the target. The front surface of the target, where the laser pulse was incident, is optically flat. It was placed on a *x* − *y* − *z* − *θ* stage assembly to enable for raster scanning such that every time the laser pulse is incident upon an undamaged part on the Cu-substrate. Defocusing was carried out by moving the target towards the target-normal-direction (x-direction). The generated particles were diagnosed using a TP (Thomson parabola spectrometer)^40^, coupled with a micro-channel-plate (MCP) and a CCD camera (shown in Fig. 1). Before entering the field region of the TP, the particle beam was extracted using a circular aperture of 250 *μ*m, placed approximately at 55 cm away from the target front surface along the target-normal. The field region (15 cm long) of the TP consists of a pair of electrode and a pair of permanent magnet in parallel combination. After the field assembly, the particle beam was allowed to drift before impinging on the MCP. The length of the field-free-region was chosen to be approximately 30 cm. The electric and magnetic fields in the TP disperse ions into parabolic traces according to their charge-to-mass ratio. Simultaneous imaging and arrival time measurements were used to detect and measure the energy of the accelerated neutral atoms (illustrated in Fig. 2). If the field (either the electric or the magnetic field) in the TP is small, ions would be dispersed in a straight line. The field was then increased until all the ions were deflected out of the detector. At this configuration, the TP-spectrogram was left only with the central bright spot. Neutral atoms and the photons unaffected by any electric field and/or magnetic field were detected in this central spot. We have used only the magnetic field to deflect the ions for neutral atom detection to avoid re-ionization of the neutrals in the field region of the spectrometer. After ensuring that the detector was exposed only to neutral particles, we measured the arrival time of the particles. Photons travel at the speed of light and reach the detector within a few nanoseconds, whereas the more massive neutral atoms arrive at the detector on a microsecond time scale. From the arrival time profile the energy spectra and the percentage of neutralization can be easily extracted.
